# Behavioral evidence for pattern separation in human episodic memory

**DOI:** 10.1101/lm.051821.120

**Published:** 2020-08

**Authors:** Ewa Zotow, James A Bisby, Neil Burgess

**Affiliations:** 1 Institute of Cognitive Neuroscience, University College London, London WC1N 3AZ, United Kingdom; 2 Institute of Neurology, University College London, London WC1N 3BG, United Kingdom; 3 Division of Psychiatry, University College London, London W1T 7BN, United Kingdom

## Abstract

An essential feature of episodic memory is the ability to recall the multiple elements relating to one event from the multitude of elements relating to other, potentially similar events. Hippocampal pattern separation is thought to play a fundamental role in this process, by orthogonalizing the representations of overlapping events during encoding, to reduce interference between them during the process of pattern completion by which one or other is recalled. We introduce a new paradigm to test the hypothesis that similar memories, but not unrelated memories, are actively separated at encoding. Participants memorized events which were either unique or shared a common element with another event (paired “overlapping” events). We used a measure of dependency, originally devised to measure pattern completion, to quantify how much the probability of successfully retrieving associations from one event depends on successful retrieval of associations from the same event, an unrelated event or the overlapping event. In two experiments, we saw that within event retrievals were highly dependent, indicating pattern completion; retrievals from unrelated events were independent; and retrievals from overlapping events were antidependent (i.e., less than independent), indicating pattern separation. This suggests that representations of similar (overlapping) memories are actively separated, resulting in lowered dependency of retrieval performance between them, as would be predicted by the pattern separation account.

An essential feature of episodic memory is the way in which similar experiences are stored and retrieved as separate memories. It is well established that the formation of new memories and the recall of old ones rely on the hippocampus, which acts as a convergence zone to bind together disparate elements from an event into a single memory engram (O'Keefe and Nadel 1978; Tulving 1983; Damasio 1989; Cohen and Eichenbaum 1993; Davachi 2006; Eichenbaum et al. 2007). Computational models of the hippocampus have long posited that, while encoding relies on pattern separation where overlapping representations are made more distinct, presentation of a partial cue will result in pattern completion and the complete retrieval of the stored representation (Marr 1971; McClelland et al. 1995; Norman and O'Reilly 2003). Accordingly, these complementary mechanisms should allow for holistic retrieval of all aspects of an event without interference from similar overlapping experiences.

High similarity of the neural patterns associated with different memories leads to decreased accuracy (Hopfield 1982; Treves and Rolls 1992). Similar memories may therefore require a mechanism which can decorrelate their representations, reducing the risk of their interference. Representations of unrelated memories, on the other hand, already produce sufficiently different neural patterns, and so their associated patterns do not need to be actively orthogonalized in this way (Leutgeb et al. 2007).

Evidence for pattern separation has mostly been derived from rodent studies in which the dentate gyrus (DG), due to its large numbers of neurons and sparse coding, is thought to support the decorrelation of input signals before reaching CA3 (Leutgeb et al. 2007). Lesions to the DG but not CA3 of rodents result in novelty detection impairments following exposure to a new spatial environment, possibly due to increased interference from a previous environment (Hunsaker et al. 2008). In contrast, the recurrent collaterals in CA3 are thought to form an autoassociative network to support pattern completion with rodents found to show impaired object location memory when the number of cues available is reduced (Nakazawa et al. 2002; Gold and Kesner 2005).

In humans, pattern separation is often inferred behaviorally from the pattern of retrieval errors. For example, the mnemonic similarity task (MST) requires participants to recognize whether a probe item is old (i.e., previously seen, as some items are repeated), similar (a new item that is similar but not identical to a previously seen item), or new, with pattern separation indexed as the successful identification of similar items (Kirwan and Stark 2007; Stark et al. 2015). The performance on this task, however, does not tell us whether the encoded representations of similar items are more separated than of dissimilar items, but instead may reflect how accurately the original items were encoded (therefore enabling detection of the novelty of similar new items). At the neural level, it is also not clear whether observed changes to lure items reflect encoding of the lure or a recall-to-reject of previously encountered items (Hunsaker and Kesner 2013). Functional neuroimaging studies using the MST sought to solve the problem of explicit recall-to-reject by using incidental encoding and found greater activity in the DG/CA3 subregion of the hippocampus during presentation of a foil compared to presentation of a previously seen item (Bakker et al. 2008; Lacy et al. 2011).

Supporting evidence came from a number of neuroimaging studies using more complex memory representations. Similar spatial environments were found to be represented with distinct hippocampal patterns (Bonnici et al. 2012; Stokes et al. 2013), and more distinct patterns predicted later recall of the layout of the environment (Kyle et al. 2015). The hippocampal activity patterns associated with overlapping routes (Chanales et al. 2017) and narratives with overlapping elements (Chadwick et al. 2011; Milivojevic et al. 2016) were found to diverge over time. However, these tasks either did not provide a behavioral read-out or did not look at memory for multielement events.

In the current study, we focused on the role of pattern separation in preventing incorrect pattern completion in episodic memory. While the elements of each event should be bound together into coherent representations, resulting in a holistic retrieval of each event, the patterns associated with different events should be sufficiently distinct to prevent interference. Recent studies have shown that multielement events (e.g., person—location—object) are stored and retrieved as single coherent representations. That is, successful retrieval of one association from an event is statistically related to retrieval of all other associations from the same event (Horner and Burgess 2013, 2014; Horner et al. 2015; Bisby et al. 2018). This within-event dependency across retrievals and the holistic manner in which events are reexperienced is consistent with pattern completion. In addition, neuroimaging evidence has shown that the individual elements from an event are represented by activity in separate neocortical regions; at retrieval all elements, including the non-cue/non-target item, are reinstated when a single association is tested, and this reinstatement is supported by the hippocampus (Horner et al. 2015).

The observed within-event dependency across retrievals of associations from events offers a potential basis to examine pattern separation processes at the behavioral level. For instance, while retrievals of associations within an event are related, retrieval success between separate unrelated events should be independent. Further, as pattern separation aims to reduce interference of overlapping memories by making their representations less similar, the retrieval success across two events that share a common element (e.g., the same object used in two separate events) should show a decreased dependency if subject to pattern separation at encoding. That is, the probability of making a successful retrieval from one event should be dependent on the retrieval of all other elements from that event but independent from retrieval success of elements from an unrelated event, and “negatively dependent” (i.e., showing decreased dependency) from a related event with which it shares an element.

Across two experiments, we aimed to assess the pattern of retrievals both within and across events for evidence of pattern separation- and pattern completion-like processes. Similar to previous work (Horner and Burgess 2013, 2014; Horner et al. 2015; Bisby et al. 2018; Joensen et al. 2019; Ngo et al. 2019), we instructed participants to encode multielement events involving a person, a location and an object. Some of the events overlapped with another event, that is, they shared a common element (e.g., the same person was part of both events). Other events were fully unique (nonoverlapping) and didn't share any elements with other events (Fig. 1). At test, associative accuracy was assessed using a six alternative forced choice test.

**Figure 1.**
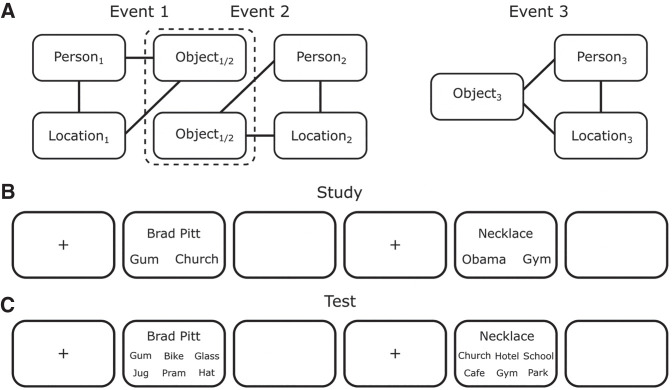
Experimental stimuli and example of a trial sequence. (*A*) Experimental stimuli: an example of the associative structure of two matched, overlapping events where the object is the common element (i.e., the same object appears in both events). Nonoverlapping events consisted of the same elements but shared no elements with other events. Example of the trial sequence and timing for (*B*) the study phase and (*C*) the test phase.

We assessed the statistical dependency of success in retrieving different associations from either the same event or from different events, where within-event dependency is thought to reflect pattern completion—a common process in retrieving, for example, both the location and the person in an event, when cued by the object (see Horner and Burgess 2013, 2014). Here we are additionally interested in the dependency between retrievals from different events as a function of whether or not they share an element. For instance, the probability of retrieving a person when cued by an object for both event_1_ and event_2_ (where event_1_ and event_2_ could be either unrelated or overlapping). The dependency measure reflected how often these associations are retrieved either both correctly or both incorrectly.

To account for the performance levels of each participant across different types of element, and levels of guessing, the dependency measure in each analysis was compared to independent and dependent models, predicting respectively the level of dependency expected from independent pairwise associations and from associations modulated by a common factor for each event (estimated from other within-event associations; Horner and Burgess 2014).

According to the pattern separation account of memory formation, the representations of overlapping events will actively separate to prevent interference, leading to reduced dependency in retrievals from one event on retrievals from the other event than that observed for unrelated, nonoverlapping events.

## Results

### Experiment 1

#### Associative accuracy

Overall accuracy across all trials was good (66.42%, SD = 17.66) and well above chance (chance would be 16.7% given the six test options). Analysis of performance across cue-type (collapsed across retrieval-type) using a 2 × 3 repeated-measures ANOVA with factors of event type (overlapping, nonoverlapping) and cue-type (person, location, object) showed a significant main effect of event type (*F*_(1,29)_ = 13.41, *P* = 0.001, *η*^2^ = 0.32) with slightly higher accuracy in overlapping events. There was no significant main effect of cue type (*F*_(2,58)_ = 2.56, *P* = 0.09, η^2^ = 0.08) nor a cue-type × event type interaction (*F*_(2,58)_ = 0.07, *P* = 0.93, *η*^2^ < 0.01). A similar 2 × 3 ANOVA on target-type (collapsed across cue-type) showed no main effect of retrieved type (*F*_(2,58)_ = 2.31, *P* = 0.11, *η*^2^ = 0.07). The main effect of event was the same as in the cue-type analysis. There was no interaction between retrieved type and event type (*F*_(2,58)_ = 1.96, *P* = 0.15, *η*^2^ = 0.06; (see Table 1).

**Table 1.**
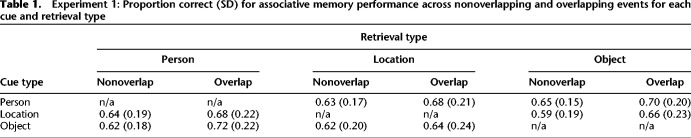
Experiment 1: Proportion correct (SD) for associative memory performance across nonoverlapping and overlapping events for each cue and retrieval type

#### Within-events dependency

Dependency was assessed by constructing contingency tables for retrieving two elements when cued with the third element, and retrieving one element when cued by the other two elements across retrieval trials (see Materials and Methods). We then calculated within-event dependency (D) in the data by taking the proportion of events where elements were both correctly or incorrectly retrieved (Table 3). This dependency was then compared to the amount of dependency predicted if retrievals from the same event were completely independent (Di) or dependent (Dd; see Materials and Methods for more information on how the models were constructed). We compared dependency in the data with both independent and dependent models separately for nonoverlapping and overlapping events. A 2 × 3 repeated-measures ANOVA with factors of event type (nonoverlapping, overlapping) and dependency measure (D, Di, Dd) showed a significant interaction between event type and dependency (*F*_(1.52,9.82)_ = 9.82, *P* = 0.001, *η*^2^ = 0.25; Greenhouse-Geisser corrected).

**Figure 2.**
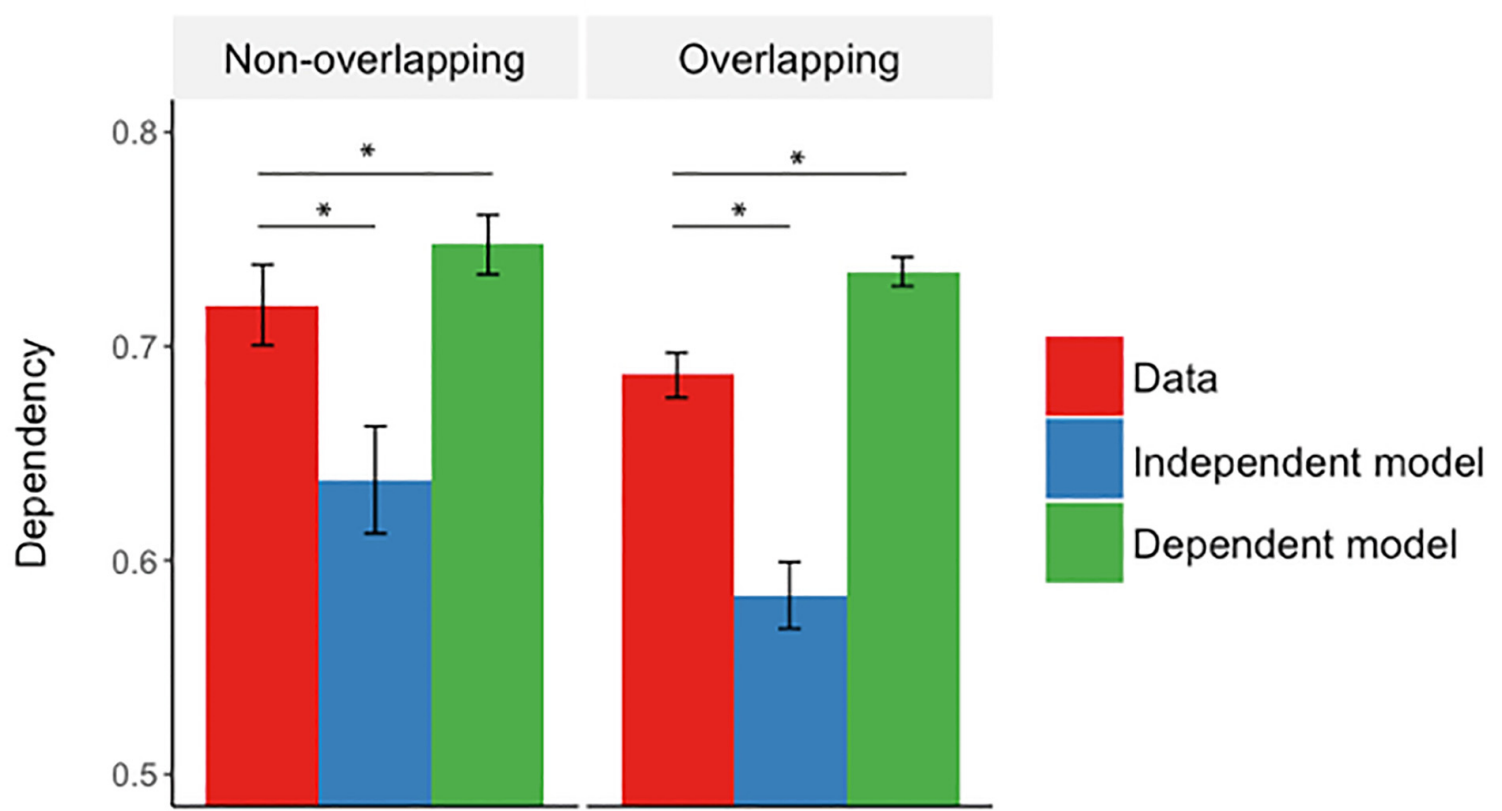
Mean dependency for the observed data, the independent model and the dependent model for the within-event analysis. The error bars represent ±1 SEM. (*) *P* < 0.001.

To further assess the interaction, we first analyzed overlapping and nonoverlapping events separately, comparing the data to independent and dependent models. For nonoverlapping events, we found greater within-event dependency in the data compared to the independent model (D > Di, *t*_(29)_ = 7.41, *P* < 0.001, *d* = 1.33) and less than the dependent model (D < Dd, *t*_(29)_ = 4.06, *P* < 0.001, *d* = 0.75). Similarly for overlapping events, dependency in the data was greater than the independent model (D > Di, *t*_(29)_ = 9.00, *P* < 0.001, *d* = 1.66) and was less than the dependent model (D < Dd, *t*_(29)_ = 10.46, *P* < 0.001, *d* = 1.67) (Fig. 2).

We next performed a direct comparison of dependency between nonoverlapping and overlapping events. We calculated a difference score between each participant's dependency and the independent model (D-Di) and between each participant's dependency and the dependent model (D-Dd). This reflected a *relative dependency* level; positive scores indicated a higher dependency level than predicted by the average performance under the relevant model. We then compared the relative dependency scores between nonoverlapping and overlapping events.

There was no difference between nonoverlapping and overlapping events in the amount of dependency relative to the independent model (D-Di, *t*_(29)_ = 1.87, *P* = 0.077, *d* = 0.34). A comparison of dependency relative to the dependent model showed a significant difference between events (D-Dd, *t*_(29)_ = 3.04, *P* = 0.005, *d* = 0.55) with less dependency in the data compared to the dependent model for overlapping events. Importantly, both nonoverlapping and overlapping events showed greater dependency in the data than predicted by the independent model.

#### Dependency in matched events

As we have proposed, successful pattern separation between events that share a common element would likely reduce dependency due to the decorrelation of neural representations of those events. To examine this prediction, we examined the amount of dependency across matched overlapping events that shared a common element (i.e., whether retrieval success of an association from one event is dependent on the retrieval success of the matching association from an overlapping event) and compared this to the amount of dependency across nonoverlapping events. Associations where the common element was the cue were not included in this analysis.

We calculated dependency in the data and corresponding independent and dependent models across pairs of matched, overlapping events (see Materials and Methods for how this was achieved).

First, we looked at whether dependency in retrievals from overlapping events differed from nonoverlapping events. We ran a 2 × 3 repeated-measures ANOVA with factors event type (nonoverlapping, overlapping) and dependency measure (D, Di, Dd). It is important to note that the measures used here are the within-event dependency measures for the nonoverlapping events and the across-events dependency measures for the overlapping events. As expected, we found a significant interaction (*F*_(1.46,42.24)_ = 37.91, *P* < 0.001, *η*^2^ = 0.57, Greenhouse–Geisser corrected).

**Figure 3.**
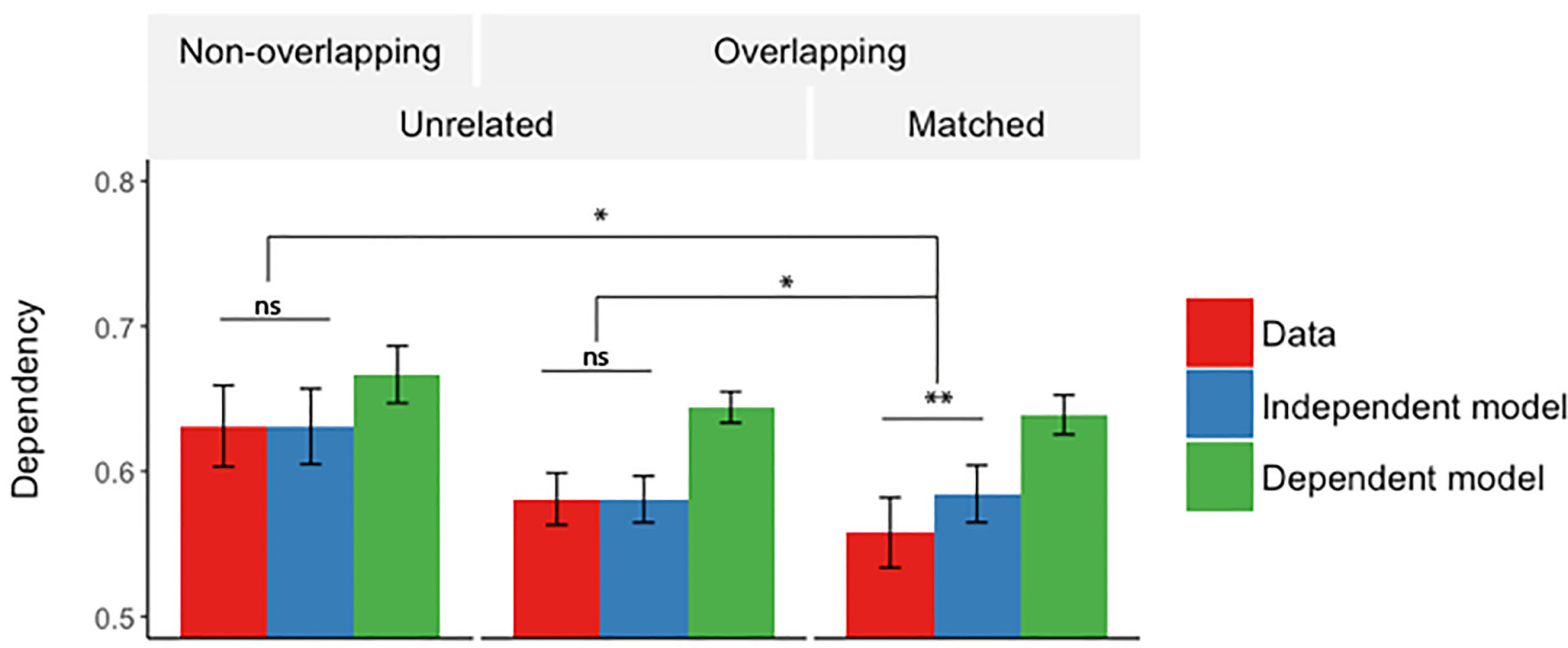
Mean across-event dependency for pairs of unrelated events (first set of three bars: unrelated, nonoverlapping events; second set of three bars: unrelated, overlapping events) and pairs of matched, overlapping events (third set of three bars). The data for unrelated pairs was obtained by randomly selecting pairs of unrelated events (separately for overlapping and nonoverlapping events) and calculating the dependency and the models, and repeating this procedure 1000 times, each time with a different set of random pairs. The *P*-values were obtained using Brown's method for combining tests of significance (Brown 1975). The bars represent the mean relative dependency, and the error bars represent the mean standard error across the 1000 tests (unrelated events) or ±1 standard error (matched events). (*) *P* < 0.01; (**) *P* < 0.001.

To test the hypothesis that the overlap leads to a lowered dependency across matched events, we compared the amount of across-event dependency to its independent model for overlapping, matched events. We tested whether the retrieval of associations from one event depends on the retrieval of associations from its matched event. As predicted, this across-events dependency was lower than the independent model (D < Di, *t*_(29)_ = 2.70, *P* = 0.011, *d* = 0.48), and lower than the dependent model (D < Dd, *t*_(29)_ = 6.48, *P* < 0.001, *d* = 1.17) (third set of three bars in Fig. 3). This pattern is different to the one observed in the within-events dependency comparisons where the dependency in data was higher than predicted by the independent model (Fig. 2). The lower than baseline (independent model) dependency supports the hypothesis that the representations of similar events are actively separated.

Across participants, the level of relative dependency also was highly correlated with the interference measure, that is, the correlation between accuracy on overlapping pairs of events (*r* = 0.80, *P* < 0.001).

Next, we directly compared the dependency between nonoverlapping and overlapping events. Here again, we used the within-event dependency measures for the nonoverlapping events and the across-events dependency measures for the overlapping events. The level of dependency relative to the independent model was lower for the overlapping events (D-Di, *t*_(29)_ = 6.90, *P* < 0.001, *d* = 1.26). A comparison of dependency relative to the dependent model also showed a significant difference between events (D-Dd, *t*_(29)_ = 4.66, *P* < 0.001, *d* = 0.85), with lower dependency as compared to the dependent model in the overlapping events.

#### Comparison of across-events relative dependencies

Crucially, we wanted to see whether the dependency score relative to the independent model for the matched pairs of events differed from the relative dependency for unrelated pairs. If matched events are stored more independently than what would be expected from unrelated memories, this would provide evidence for pattern separation of overlapping episodic memories. To test this, we randomly paired unrelated events (separately for events of nonoverlapping and overlapping type). Unrelated events of overlapping type were the events that did have a matched pair, but here were paired up with another (unrelated) event to calculate the across-events dependency. For example, if event_1_ overlapped with event_2_, in this analysis it could be randomly paired with any other event from the set of overlapping paired events (e.g., event_3_) but not with the event_2_. This created pairs of events that were of unrelated, overlapping type with no shared elements (in contrast to pairs of matched events which shared one common element with each other).

We then calculated the across-events dependency measures for these pairs in the same way as for the matched pairs of events (see above). This was to ensure that the lowered dependency is not a result of the event type, but rather that it is specific to pairs of matched events with a common element. In other words, we wanted to see whether the need for active separation of two related episodes leads to a “negative” relative dependency between them.

As each analysis used a randomly created set of unrelated event pairs, the specific pairs selected in any given analysis could have affected the results. We therefore ran each analysis 1000 times and used a Brown's method (i.e., an extension to the Fisher's combined probability test; Brown 1975; Poole et al. 2016) which provides a combined *P*-value for nonindependent tests.

Each analysis compared the relative dependency in the matched pairs (which was the same in all 1000 analyses, as these pairs were not randomly selected) to the relative dependency in the same number of unrelated pairs of events, with different unrelated pairs selected each time.

As would be predicted by a pattern separation account, the matched events showed lower relative dependency than unrelated events of nonoverlapping type, *P* < 0.008, and of overlapping type, *P* < 0.006 (Fig. 3). The two types of unrelated events (i.e., chosen from overlapping or nonoverlapping events) were not significantly different from each other, *P* = 0.532, and neither was significantly different from their baseline, i.e., the independent model (*P* = 0.497 for unrelated nonoverlapping events and *P* = 0.475 for unrelated overlapping events; Fig. 3).

### Experiment 2

Experiment 1 compared the dependency in retrieval of associations from events that were fully unique or that shared a common element with another event (i.e., overlapping events). The results were consistent with pattern separation of overlapping memories. Experiment 2 set out to replicate these behavioral results while measuring brain activity in an fMRI task (not reported here).

#### Associative accuracy

Overall accuracy was slightly lower than in Experiment 1, at 54.85% (SD = 19.43), but still well above the chance level of 16.7%. Analysis of performance across cue-types (collapsed across retrieval-types) using a one-way repeated-measures ANOVA showed no effect of cue type (*F*_(2,58)_ = 0.03, *P* = 0.967, *η*^2^ = 0.001), and analysis of performance across retrieved-type (collapsed across cue-type) also showed no effect of retrieved-type (*F*_(2,58)_ = 1.60, *P* = 0.212, *η*^2^ = 0.09). The means and standard deviations for accuracy across different cue and retrieval types for both conditions are in Table 2.

**Table 2.**
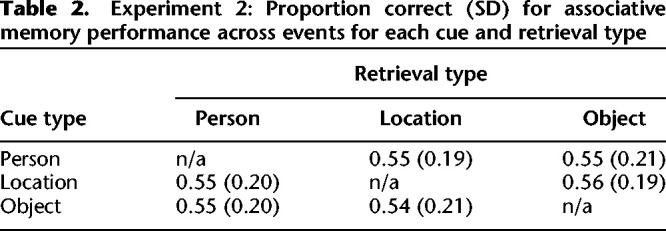
Experiment 2: Proportion correct (SD) for associative memory performance across events for each cue and retrieval type

#### Within-events dependency

As in Experiment 1, the within-events dependency was assessed by constructing contingency tables for retrieving two elements when cued with the third element, and retrieving one element when cued by the other two elements across retrieval trials (see Materials and Methods of Experiment 1). The within-event dependency (D) in the data was calculated as the proportion of events where elements were both correctly or incorrectly retrieved. This dependency measure was then compared to the amount of dependency predicted if retrievals from the same event were completely independent (Di) or dependent (Dd; see Materials and Methods of Experiment 1 for more information on how the models were constructed).

**Figure 4.**
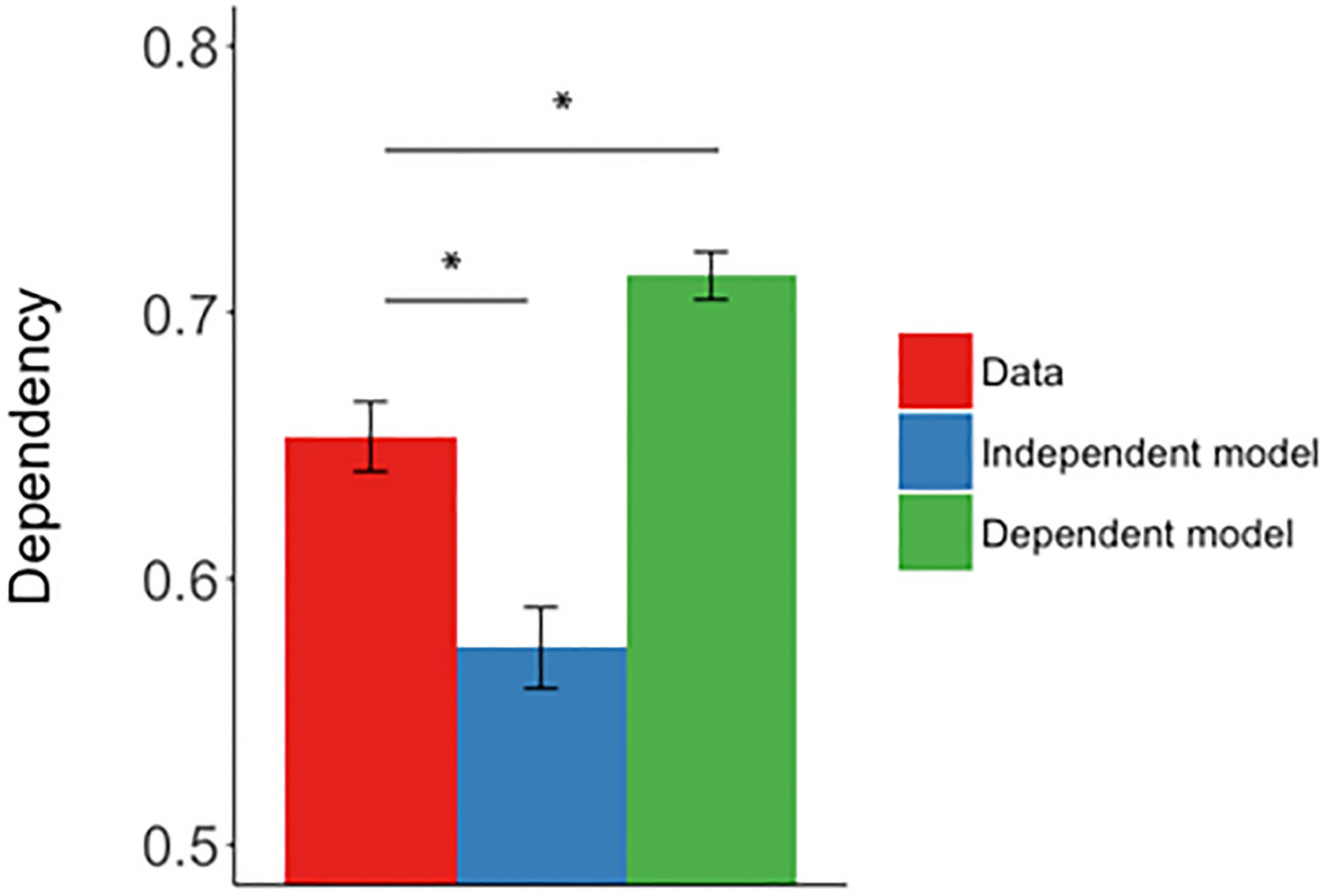
Mean dependency for the observed data, the independent model and the dependent model for the within-event analysis. The error bars represent ±1 SEM. (*) *P* < 0.001.

The within-event dependency in the data was greater than the independent model (D > Di, *t*_(29)_ = 7.38, *P* < 0.001, *d* = 1.35) and lower than the dependent model (D < Dd, *t*_(29)_ = 11.09, *P* < 0.001, *d* = 2.02) (Fig. 4).

#### Dependency in matched events

As in Experiment 1, we examined the amount of dependency across matched overlapping events that shared a common element (i.e., whether retrieval success of an element from one event is dependent on the retrieval success of an element from an overlapping event). We calculated dependency in the data and corresponding independent and dependent models across pairs of events (see Materials and Methods of Experiment 1).

**Figure 5.**
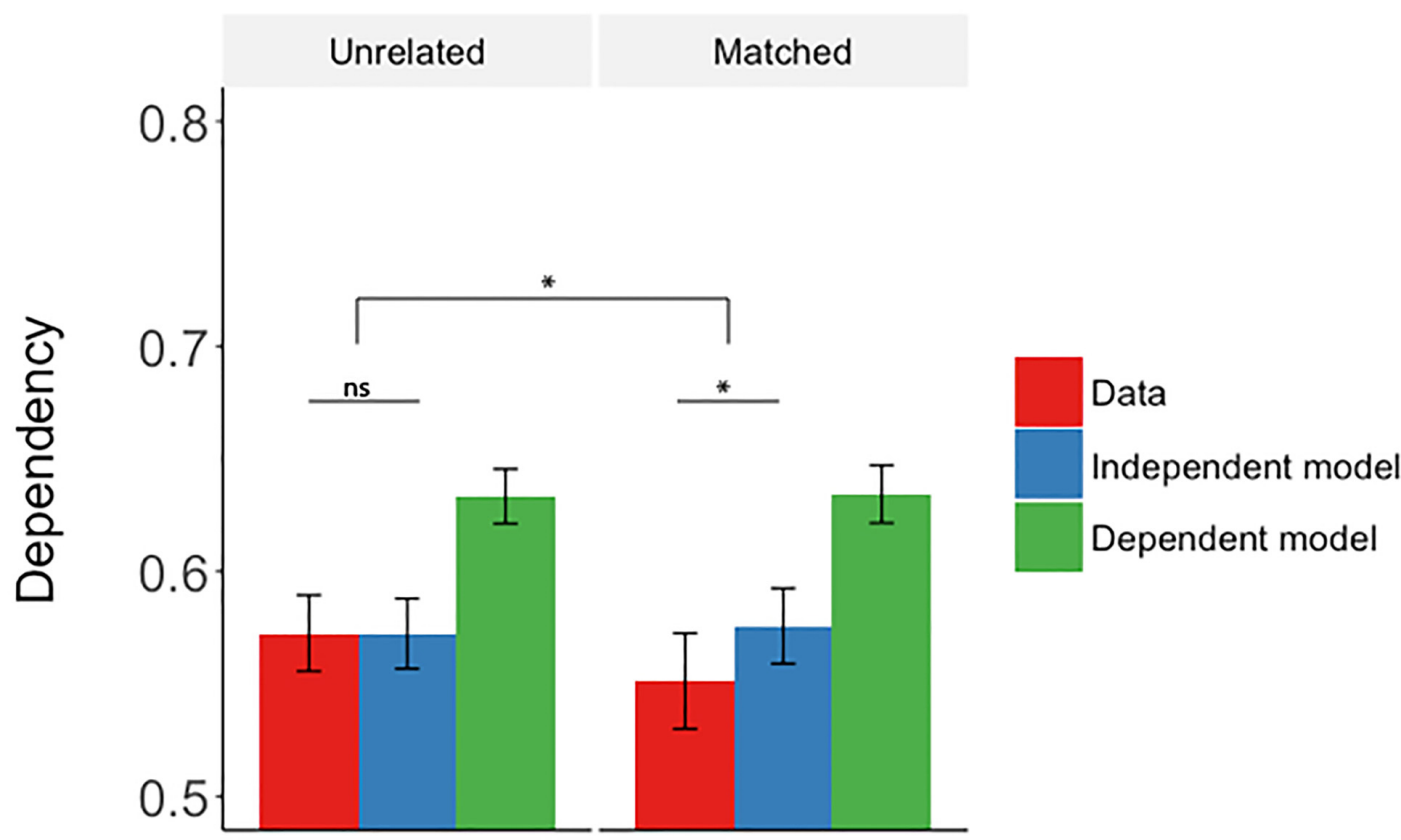
Mean across-event dependency for pairs of unrelated events and pairs of matched, overlapping events. The data for unrelated pairs was obtained by randomly selecting pairs of unrelated events and calculating the dependency and the models, and repeating this procedure 1000 times, each time with a different set of random pairs. The *P*-values were obtained using Brown's method for combining tests of significance (Brown 1975). The bars represent the mean relative dependency, and the error bars represent the mean standard error across the 1000 tests (unrelated events) or ±1 standard error (matched events). (*) *P* = 0.001.

Consistently with the behavioral study, the across-events dependency was lower than the independent model (D < Di, *t*_(29)_ = 3.04, *P* = 0.001, *d* = 0.56), and lower than the dependent model (D < Dd, *t*_(29)_ = 8.54, *P* < 0.001, *d* = 1.56) (Fig. 5).

#### Comparison of across-events relative dependencies

As in Experiment 1, we calculated a relative dependency measure (difference score between each participant's dependency and its independent model: D-Di). We looked at whether the relative dependency score is lower for overlapping events than for pairs of unrelated events. As before, in order to create unrelated pairs, we randomly selected sets of two events with no shared elements. We then calculated the across-events dependency measures for these pairs in the same way as for the matched pairs of events (see Materials and Methods of Experiment 1). Because each analysis selected a random set of unrelated pairs, we repeated the procedure 1000 times, with different unrelated pairs selected each time, and used the Brown's method to combine the probability values (Brown 1975; Poole et al. 2016).

Supporting the results from Experiment 1, we found that overlapping events had lower relative dependency than the unrelated events (*P* = 0.001). The dependency in unrelated events was not different from baseline, i.e., the independent model (*P* = 0.289) (Fig. 5).

## Discussion

Computational models of hippocampal function propose that similar memories are stored as distinct nonoverlapping representations through a process of pattern separation (Marr 1971). This process is complementary to pattern completion by which a subset of cues from a previous experience can reactivate the stored pattern representing that experience (Marr 1971; Hopfield 1982; McClelland et al. 1995; Rolls 2015). These processes therefore give rise to two complementary characteristics of memory such that even similar events are stored as independent nonoverlapping representations, and that all elements of an event can be retrieved in a holistic manner. To test these hypotheses, we examined whether events that share a common element would be represented as more distinct than unrelated events, as assessed by the relationships between retrievals from within and between the overlapping or unrelated events.

Since the role of pattern separation at encoding is to reduce interference during pattern completion at retrieval, we adapted a pattern completion task to provide a measure of both processes. Two experiments provided consistent results. While retrievals from within the same event show increased statistical dependency (as predicted by pattern completion), retrievals from overlapping events showed lower dependency than expected from unrelated events. This supports the proposal that pattern separation serves to decorrelate similar inputs by transforming their representations into orthogonal patterns (Marr 1971; McClelland et al. 1995).

Our results are consistent with the view that events are stored as coherent representations and retrieved in a holistic manner. In accordance with previous studies, we found greater dependency in the retrieval of elements from the same event (Horner and Burgess 2013, 2014; Horner et al. 2015; Bisby et al. 2018), supporting the holistic way in which episodic memories are stored and retrieved (Figs. 2, 4; Tulving 1983). We assume that within-event dependency reflects the associative structure in which event elements are bound together into single representation. When cued by a single event element, all associated within-event elements are reinstated. This is consistent with computational models of hippocampal pattern completion and the way event elements are stored in an autoassociative network in which presentation of a partial cue will cause reinstatement of all associated event elements (Marr 1971; Hopfield 1982; McClelland et al. 1995). Neuroimaging evidence, using a similar task and dependency measure as we used here, is also complementary to our results in suggesting that all within-event elements are reinstated in neocortical areas and this reinstatement is supported by the hippocampus (Horner et al. 2015) and more specifically, region CA3 (Grande et al. 2019).

Within-event dependency was also seen in events even when they overlapped with other events (i.e., they shared a common element). This finding is important as it suggests that, while pattern separation should specifically affect representations that may interfere with each other due to their similarity (i.e., representations of overlapping events), the constituent elements of each of these events, which needs to be bound into coherent narratives, are not separated but instead continue to show pattern completion. This is consistent with findings from rats which showed that the hippocampus binds information encountered in the same context together while separating events from different contexts (Wills et al. 2005; McKenzie et al. 2014).

While retrieval of within-event elements should show high dependency, we should not observe the same pattern of results in across-event dependency (i.e., the extent to which retrieving an association from one event depends on the retrieval of associations from another event). If events are represented as separate engrams, we would expect that the dependency in the retrieval of their respective associations will be independent, that is, as predicted by the independent model. For unrelated pairs of events this was indeed found to be the case (Figs. 3, 5).

Interestingly, and crucially for our hypothesis, retrievals across overlapping events (i.e., events with one element in common) showed a level of dependency that was significantly lower than predicted by the independent model (Figs. 3, 5). For dependency to be less than expected by the independent model, the probability of retrieving an association from one event must be negatively related to the probability of retrieving an association from its overlapping event. Although overlapping events shared an element and so might be expected to become associated during learning, the success on the task depended on the ability to discriminate between the two events in order to avoid interference, which we propose is achieved by an increased separation of their respective representations. The finding that the dependency was “negative” only for the matched pairs from overlapping events, but not for pairs from unrelated overlapping events, suggests that the results are not simply due to the fact that events that are not fully unique are processed in a different way; rather, the increased separation occurs only for the specific pairs of events which share an element in common and not for all events that happen to share a common element with another event. The role of pattern separation may be therefore to accentuate the differences between similar input patterns, as distinct (unrelated) memories already produce sufficiently different output and do not suffer from the same interference (Leutgeb et al. 2004; Vazdarjanova and Guzowski 2004; Lacy et al. 2011).

A potential alternative explanation for reduced dependency across overlapping events could be retrieval induced forgetting, where retrieval of an association with a shared element impairs later retrieval of the paired association (i.e., the association with the same element) from the other event. To address this, in a post-hoc analysis we analyzed the dependency between retrievals of matched associations from overlapping events that did not include a common item, that is, dependency between retrievals of associations A1–B1 and A2–B2 (tested in either order), where the two overlapping events comprised items A1,B1,C and A2,B2,C. We found that the dependency between these associations from overlapping events was significantly lower than for corresponding associations between unrelated events, despite there being relatively few trials for this analysis (*P* = 0.015, *d* = 0.40, combined across both experiments using Brown's method as described in Materials and Methods).

The high dependency within events and lowered dependency across overlapping events are consistent with the proposed role of the different hippocampal mechanisms in storage of episodic memories (e.g., McNaughton and Morris 1987). The autoassociative network of the CA3 is proposed to store learnt associations in its recurrent connections, which allows for linking all elements of a memory together and for their reinstatement through pattern completion at retrieval. This underlies the holistic retrieval of all memory elements, as suggested by the high within-events dependency in the current study. While event-elements are bound together, different events are stored as distinct memory engrams. However, as neural patterns associated with different stored memories become more similar, the retrieval accuracy falls (Hopfield 1982; Amit et al. 1987; Treves and Rolls 1992), and so there is a need for a mechanism that actively decorrelates similar inputs to avoid subsequent interference. This is performed by the dentate gyrus which decorrelates the representations of similar memories by “selecting” a different population of CA3 neurons for their storage, driving their respective representations further apart (Marr 1971; Treves et al. 2008). As a result, similar memories have less similar representations than unrelated memories do on average. These two mechanisms ensure that the overlapping representations are less likely to interfere with each other while their constituent elements are bound together into single representations. This shows the complementary functions of pattern separation and completion in supporting episodic memory.

Our paradigm extends upon research examining pattern separation processes in humans by using a task that segregates the encoding and retrieval phases, and distinguish between successful encoding and pattern separation. Pattern separation and pattern completion operate at, respectively, encoding and retrieval, and so it has been suggested that an appropriate task should be one where the process of interest (separation or completion) is the most appropriate strategy (Hunsaker and Kesner 2013; Liu et al. 2016). The continuous version of the MST requires “recall-to-reject” in addition to intentional encoding, making it difficult to separate the two processes, an issue recognized by the authors (Kirwan and Stark 2007). The study-test version, although separates encoding and retrieval, still does not examine whether the encoded representations of similar items are more separated than those of dissimilar items, and may instead reflect the accuracy of encoding of the previously seen items.

Our findings are consistent with a study where similar scenes were paired with either two different facial stimuli, and so needed to be discriminated, or with the same face. The demand to distinguish between the similar scenes led to reduced interference of those scenes in a subsequent association task with novel stimuli (Favila et al. 2016). The need for discrimination and the subsequent reduction in interference were both related to decreased similarity in the scenes’ hippocampal representations assessed by fMRI. This is consistent with the view that pattern separation serves to reduce interference, in the context of separation of similar static scenes rather than the multielement events studied here. The current task provides another way of looking at behavioral pattern separation while overcoming some of the limitations of pattern separation/completion tasks outlined by Liu et al. (2016) and others (Kirwan and Stark 2007; Hunsaker and Kesner 2013).

The current task targets the associative role of the hippocampus in multielement episodic memory. The hippocampus is thought to be specifically required for contextual or relational memory as opposed to memory for individual items or a feeling of familiarity (Uncapher and Rugg 2005; Horner et al. 2012; Aggleton and Brown 1999). Item memory, on the other hand, may be processed in other medial temporal lobe structures (e.g., perirhinal cortex) (McClelland et al. 1995; Eichenbaum et al. 2007), but see also (Squire and Zola-Morgan 1991). Although the use of an old/new recognition paradigm with similar foils may require the hippocampus for recollection of small details rather than familiarity (Holdstock et al. 2002), it does not require pattern separation at encoding and so does not directly address the formation of separated representations for storage to avoid interference during recall.

In conclusion, we present a novel and relatively process-pure way of behaviorally investigating pattern separation. We propose that the lowered (“negative”) dependency in retrieval of associations from overlapping events results from the need to differentiate their neural representations through pattern separation. This finding is specific to pairs of events with a common element and not to pairs of unrelated events (whose retrieval was found to be independent from each other), which confirms the predictions regarding how similar episodic memories are represented.

## Materials and Methods

### Experiment 1

#### Participants

Thirty-nine participants were recruited from the University College London student population. Seven participants were excluded due to poor overall task accuracy (below 25%) and two due to too high accuracy (above 90%); very high performance resulted in too low variability to give reliable results in the dependency analysis (see below). The remaining 30 participants (25 females, 5 males; sample size exceeds related behavioral studies, Horner and Burgess 2013, 2014) had a mean age of 25.61 (SD = 6.22), had normal or corrected-to-normal vision, were fluent English speakers and were familiar with Western culture including major celebrities and politicians (self-reported). The study was approved by the UCL Research Ethics Committee and all participants provided written informed consent prior to taking part in the study. Following completion of the test, participants were debriefed and paid for their time.

#### Materials

Word stimuli included 40 famous people (e.g., Tom Cruise, Barack Obama), 40 locations (e.g., supermarket, kitchen) and 40 objects (e.g., necklace, bottle). Before attending the study, participants selected famous people they were familiar with from a list of 60 candidates; out of all familiar to the participant, 40 were chosen at random (all participants were familiar with at least 40 people from the full list). Stimuli were combined to create 45 three-element events with each event consisting of a person, location, and object. Fifteen of these events were unique from each other in that they shared no common elements (i.e., nonoverlapping events; Event 3 in Fig. 1A). The remaining 30 events were combined to create 15 sets of two events in which the two events of a set shared one common element (i.e., overlapping events; Events 1 and 2 in Fig. 1A). Pairs of overlapping events with a shared element will be referred to as matched events. For matched events, the common element (person, location, or object) was counterbalanced across event sets. Novel randomized events were created for each participant. The Cogent 2000 toolbox (www.fil.ion.ucl.ac.uk) for Matlab R2016a (MathWorks) was used for stimulus presentation and data collection. We used white text on gray background, and Helvetica font of size 30.

#### Procedure

The experiment consisted of a study phase and a test phase (Fig. 1B,C). During the study phase, participants encoded a total of 45 event triplets with each comprising a person, location and object. Each trial began with a 0.5 sec fixation period, after which participants were presented with one of the event triplets (Fig. 1A). All three elements remained on the screen for 8 sec and participants were instructed to imagine all event elements interacting as vividly as possible. The screen location of each element type (person, location, object) was randomized across encoding trials. The trial ended with a blank screen presented for 1.5 sec. Events were only shown once during the study phase.

At test, associative accuracy was assessed using a six alternative forced choice test. On each trial, following a 0.5 sec fixation, participants were presented with one of the previously encoded items at the top of the screen (cue) and six possible “target” items were presented below (Fig. 1B). They were then instructed to decide which of the targets had previously appeared in the same event as the cue. All targets on a single test trial were of the same category and all appeared in the study phase as elements of different events. For example, if cueing with the person to retrieve the associated location, all six options were of locations. Participants were given 8 sec to select their option via button press. All possible cue-target pairs were tested in both directions (e.g., cue with the person to retrieve the location, cue with the location to retrieve the person), giving a total of 270 trials. The order of trials was randomized. Each trial terminated with a 1.5 sec blank screen.

#### Analysis of within-event dependency

We created 2 × 2 contingency tables for each participant. For within-event dependency, the contingency tables were based on (i) the probability of retrieving two items from the same event when cued by the remaining item from that event (AbAc; e.g., retrieving either a person “b” or a location “c” when cued by an object “A”), and (ii) the probability of retrieving an item when cued by the two remaining items from the event (BaCa; e.g., retrieving a person “a” when cued either by a location “B” or an object “C”).

This measure was then compared to an independent model and a dependent model calculated individually for each participant. This within-subject comparison accounted for individual differences in performance on the raw dependency score. The models estimated the level of dependency based on the average performance and level of guessing. The independent model assumed that the retrieval of any two elements from the same event is completely independent and is contingent only on the overall accuracy level. It was calculated by multiplying the probabilities of separately retrieving two elements from the categories in question.

The dependent model additionally adjusted the predicted level of dependency by an event-specific “episodic factor”—a measure of the average performance on a given event (across all other retrieval trials for that event) relative to the overall performance. The probability of retrieving any association from an event was weighted by the episodic factor for that event. The dependent model also accounted for the level of guessing; the episodic factor affected the probability of intentional retrieval but not the probability of correct guessing, which is assumed to be independent (see Table 3).

**Table 3.**
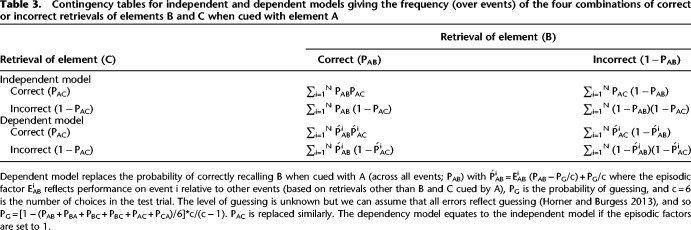
Contingency tables for independent and dependent models giving the frequency (over events) of the four combinations of correct or incorrect retrievals of elements B and C when cued with element A

For each participant, we calculated the measure of dependency as well as the independent and dependent models separately for events of nonoverlapping and overlapping types. This was to check whether both types are retrieved as complete events (i.e., both show high within-event dependency).

#### Analysis of across-event dependency

The contingency tables for dependencies across pairs of matched events were based on the probability of correctly or incorrectly retrieving associations from both events. Unlike in the “standard” within-event dependency described above, where both compared associations (e.g., Ab and Ac) came from the same event, each association came from a different event from a pair (event_1_ and event_2_). Importantly, for calculation of the dependency measure and models we excluded the trials in which the overlapping element was a cue, as these types of trials had two possible correct answers, and potentially a retrieval of the incorrect pair could interfere with the retrieval of the appropriate element. Here the contingency tables were based on (i) how the probability of retrieving a specific association depends on the probability of retrieving the same type of association from the corresponding paired event (Ab_1_Ab_2_; where A is not the shared item), (ii) how the retrieval of an item from one event depends on the retrieval of a different-type item from the corresponding event when cued by the same-type items (Ab_1_Ac_2_; where A is not the shared item), and (iii) the probability of retrieval of the same-type items when cued by different-type items in each event (Ba_1_Ca_2_; where A is the shared item, because pairs where the shared element is the cue were excluded).

### Experiment 2

#### Participants

Thirty-three neurologically healthy participants were recruited from the student population at the University College London. Three participants were excluded due to low performance (<20% accuracy). The accuracy threshold was lowered to reflect the overall lower performance in this study, potentially caused by the different setup (sitting at a desk versus lying in an fMRI scanner). The remaining 30 participants (10 males) had a mean age 24.43 (SD = 3.87), normal or corrected-to-normal vision and were right handed. Participants were paid £20 for the scanning session. The study was approved by the University College London Research Ethics Committee (1825/003) and informed consent was obtained before the session. Following completion of the test, participants were debriefed and paid for their time.

#### Materials

The same stimuli as in Experiment 1 were used. As shown above, the dependency results for pairs of unrelated events were the same regardless of whether those pairs consisted of fully unique events or events which shared a common element with another event. Therefore, in this experiment only events of overlapping type were used, allowing for the use of a higher number of overlapping pairs while minimizing the overall time in the scanner. Word stimuli included 30 famous people (e.g., Tom Cruise, Barack Obama), 30 locations (e.g., supermarket, kitchen), and 30 objects (e.g., necklace, bottle). Participants selected famous people they were familiar with from a list of 60 candidates before attending the session; out of all familiar to the participant people, 30 were chosen at random (all participants were familiar with at least 30 people from the full list). Stimuli were combined to create 36 three-element events, giving 18 pairs with an overlapping element. The type of the common element was counterbalanced across event sets. Novel randomized events were created for each participant.

#### Procedure

Procedure was similar to Experiment 1, however as a result of this task being performed in an MRI scanner, the following aspects were changed: participants lay in a supine position with the visual display reflected from a back projector by a mirror attached to the head coil. During the test phase, participants responded using three keys with their right hand and three keys with their left hand.

The main part of the procedure was the same as in Experiment 1 and consisted of a study phase and a test phase. The study phase was the same as in Experiment 1 except it consisted of 36 trials. The test phase was also the same except it was divided into two scanning blocks with 108 trials in each. Pairwise associations for each event were split so that half were tested in the first run and half in the second run. The order within each run was randomized.

#### Analysis

All analyses of behavioral data were the same as described in Experiment 1, with the exception that here only events of overlapping type were used.

## References

[LM051821ZOTC01] Aggleton J, Brown MW. 1999 Episodic memory, amnesia, and the hippocampal, anterior thalamic axis. Behav Brain Sci 22: 425–444. 10.1017/S0140525X99002034 11301518

[LM051821ZOTC1] Amit DJ, Gutfreund H, Sompolinsky H. 1987 Information storage in neural networks with low levels of activity. Phys Rev A 35: 2293 10.1103/PhysRevA.35.2293 9898407

[LM051821ZOTC2] Bakker A, Kirwan BC, Miller M, Stark CEL. 2008 Pattern separation in the human hippocampal CA3 and dentate gyrus. Science 319: 1640–1642. 10.1126/science.1152882 18356518 PMC2829853

[LM051821ZOTC3] Bisby JA, Horner AJ, Bush D, Burgess N. 2018 Negative emotional content disrupts the coherence of episodic memories. J Exp Psychol Gen 147: 243–256. 10.1037/xge0000356 28910126 PMC5784934

[LM051821ZOTC4] Bonnici HM, Kumaran D, Chadwick MJ, Weiskopf N, Hassabis D, Maguire EA. 2012 Decoding representations of scenes in the medial temporal lobes. Hippocampus 22: 1143–1153. 10.1002/hipo.20960 21656874 PMC3470919

[LM051821ZOTC5] Brown MB. 1975 A method for combining non-independent, one-sided tests of significance. Biometrics 31: 987–992. 10.2307/2529826

[LM051821ZOTC6] Chadwick MJ, Hassabis D, Maguire EA. 2011 Decoding overlapping memories in the medial temporal lobes using high-resolution fMRI. Learn Mem 8: 742–746. 10.1101/lm.023671.111 PMC3222891 22086391

[LM051821ZOTC7] Chanales AJH, Oza A, Favila SE, Kuhl BA. 2017 Overlap among spatial memories triggers divergence of hippocampal representations. Curr Biol 27: 1–47. 10.1101/099226 28736170 PMC5576038

[LM051821ZOTC8] Cohen NJ, Eichenbaum H. 1993 Memory, amnesia, and the hippocampal system. Clin Neurobiol Hippocampus An Integr view 55173 10.1093/acprof:oso/9780199592388.003.0003

[LM051821ZOTC9] Damasio AR. 1989 The brain binds entities and events by multiregional activation from convergence zones. Neural Comput 1: 123–132. 10.1162/neco.1989.1.1.123

[LM051821ZOTC10] Davachi L. 2006 Item, context and relational episodic encoding in humans. Curr Opin Neurobiol 16: 693–700. 10.1016/j.conb.2006.10.012 17097284

[LM051821ZOTC11] Eichenbaum H, Yonelinas AP, Ranganath C. 2007 The medial temporal lobe and recognition memory. Annu Rev Neurosci 23: 123–152. 10.1146/annurev.neuro.30.051606.094328 PMC2064941 17417939

[LM051821ZOTC12] Favila SE, Chanales AJH, Kuhl BA. 2016 Experience-dependent hippocampal pattern differentiation prevents interference during subsequent learning. Nat Commun 7: 1–10. 10.1038/ncomms11066 PMC4820837 27925613

[LM051821ZOTC13] Gold AE, Kesner RP. 2005 The role of the CA3 subregion of the dorsal hippocampus in spatial pattern completion in the rat. Hippocampus 15: 808–814. 10.1002/hipo.20103 16010664

[LM051821ZOTC14] Grande X, Berron D, Horner AJ, Bisby JA, Düzel E, Burgess N. 2019 Holistic recollection via pattern completion involves hippocampal subfield CA3. J Neurosci 39: 8100–8111. 10.1523/JNEUROSCI.0722-19.2019 31405925 PMC6786823

[LM051821ZOTC15] Holdstock JS, Mayes AR, Roberts N, Cezayirli E, Isaac CL, Reilly RCO, Norman KA. 2002 Under what conditions is recognition spared relative to recall after selective hippocampal damage in humans? Hippocampus 351: 341–351. 10.1002/hipo.10011 12099485

[LM051821ZOTC16] Hopfield J. 1982 Neural networks and physical systems with emergent collective computational abilities. Proc Natl Acad Sci 79: 2554–2558. 10.1073/pnas.79.8.2554 6953413 PMC346238

[LM051821ZOTC17] Horner AJ, Burgess N. 2013 The associative structure of memory for multi-element events. J Exp Psychol Gen 142: 1370–1383. 10.1037/a0033626 23915127 PMC3906803

[LM051821ZOTC18] Horner AJ, Burgess N. 2014 Pattern completion in multielement event engrams. Curr Biol 24: 988–992. 10.1016/j.cub.2014.03.012 24746796 PMC4012134

[LM051821ZOTC19] Horner AJ, Gadian DG, Fuentemilla L, Jentschke S, Vargha-Khadem F, Duzel E. 2012 A rapid, hippocampus-dependent, item-memory signal that initiates context memory in humans. Curr Biol 22: 2369–2374. 10.1016/j.cub.2012.10.055 23177479 PMC3661975

[LM051821ZOTC20] Horner AJ, Bisby JA, Bush D, Lin W-J, Burgess N. 2015 Evidence for holistic episodic recollection via hippocampal pattern completion. Nat Commun 6: 7462 10.1038/ncomms8462 26136141 PMC4506995

[LM051821ZOTC21] Hunsaker MR, Kesner RP. 2013 The operation of pattern separation and pattern completion processes associated with different attributes or domains of memory. Neurosci Biobehav Rev 37: 36–58. 10.1016/j.neubiorev.2012.09.014 23043857

[LM051821ZOTC22] Hunsaker MR, Rosenberg JS, Kesner RP. 2008 The role of the dentate gyrus, CA3a, b, and CA3c for detecting spatial and environmental novelty. Hippocampus 18: 1064–1073. 10.1002/hipo.20464 18651615

[LM051821ZOTC23] Joensen BH, Gaskell MG, Horner AJ. 2019 United we fall: all-or-none forgetting of complex episodic events. J Exp Psychol Gen 149: 230–248. 10.1037/xge0000648 31305093 PMC6951107

[LM051821ZOTC24] Kirwan BC, Stark CEL. 2007 Overcoming interference: an fMRI investigation of pattern separation in the medial temporal lobe. Learn Mem 14: 625–633. 10.1101/lm.663507 17848502 PMC1994079

[LM051821ZOTC25] Kyle CT, Stokes JD, Lieberman JS, Hassan AS, Ekstrom AD. 2015 Successful retrieval of competing spatial environments in humans involves hippocampal pattern separation mechanisms. Elife 4: e10499 10.7554/eLife.10499 26613414 PMC4733045

[LM051821ZOTC26] Lacy JW, Yassa MA, Stark SM, Muftuler LT, Stark CEL. 2011 Distinct pattern separation related transfer functions in human CA3/dentate and CA1 revealed using high-resolution fMRI and variable mnemonic similarity. Learn Mem 18: 15–18. 10.1101/lm.1971111 21164173 PMC3023966

[LM051821ZOTC27] Leutgeb S, Leutgeb JK, Treves A, Moser M-B, Moser EI. 2004 Distinct ensemble codes in hippocampal areas CA3 and CA1. Science 305: 1295–1298. 10.1126/science.1100265 15272123

[LM051821ZOTC28] Leutgeb JK, Leutgeb S, Moser M-B, Moser EI. 2007 Pattern separation in the dentate gyrus and CA3 of the hippocampus. Science 315: 961–966. 10.1126/science.1135801 17303747

[LM051821ZOTC29] Liu KY, Gould RL, Coulson MC, Ward EV, Howard RJ. 2016 Tests of pattern separation and pattern completion in humans - a systematic review. Hippocampus 26: 207–217. 10.1002/hipo.22561 26663362

[LM051821ZOTC30] Marr D. 1971 Simple memory: a theory for archicortex. Philos Trans R Soc Lond B Biol Sci 262: 23–81. 10.1098/rstb.1971.0078 4399412

[LM051821ZOTC31] McClelland JL, McNaughton BL, O'Reilly RC. 1995 Why there are complementary learning systems in the hippocampus and neocortex: insights from the successes and failures of connectionist models of learning and memory. Psychol Rev 102: 419–457. 10.1037/0033-295X.102.3.419 7624455

[LM051821ZOTC32] McKenzie S, Frank AJ, Kinsky NR, Porter B, Rivière PD, Eichenbaum H. 2014 Hippocampal representation of related and opposing memories develop within distinct, hierarchically organized neural schemas. Neuron 83: 202–215. 10.1016/j.neuron.2014.05.019 24910078 PMC4082468

[LM051821ZOTC33] McNaughton BL, Morris RGM. 1987 Hippocampal synaptic enhancement and information storage within a distributed memory system. Trends Neurosci 10: 408–415. 10.1016/0166-2236(87)90011-7

[LM051821ZOTC34] Milivojevic B, Varadinov M, Grabovetsky AV, Collin SHP, Doeller CF. 2016 Coding of event nodes and narrative context in the hippocampus. J Neurosci 36: 12412–12424. 10.1523/JNEUROSCI.2889-15.2016 27927958 PMC6601969

[LM051821ZOTC35] Nakazawa K, Quirk MC, Chitwood RA, Watanabe M, Yeckel MF, Sun LD, Kato A, Carr CA, Johnston D, Wilson MA. 2002 Requirement for hippocampal CA3 NMDA receptors in associative memory recall. Science 297: 211–218. 10.1126/science.1071795 12040087 PMC2877140

[LM051821ZOTC36] Ngo CT, Horner AJ, Newcombe NS, Olson IR. 2019 Development of holistic episodic recollection. Psychol Sci 30: 1696–1706. 10.1177/0956797619879441 31672085 PMC7137142

[LM051821ZOTC37] Norman KA, O'Reilly RC. 2003 Modeling hippocampal and neocortical contributions to recognition memory: a complementary-learning-systems approach. Psychol Rev 110: 611–646. 10.1037/0033-295X.110.4.611 14599236

[LM051821ZOTC38] O'Keefe J, Nadel L. 1978 The hippocampus as a cognitive map. Clarendon Press, Oxford.

[LM051821ZOTC39] Poole W, Gibbs DL, Shmulevich I, Bernard B, Knijnenburg TA. 2016 Combining dependent P-values with an empirical adaptation of Brown's method. Bioinformatics 32: i430–i436. 10.1093/bioinformatics/btw438 27587659 PMC5013915

[LM051821ZOTC40] Rolls ET. 2015 Pattern separation, completion, and categorisation in the hippocampus and neocortex. Neurobiol Learn Mem 129: 4–28. 10.1016/j.nlm.2015.07.008 26190832

[LM051821ZOTC041] Squire LR, Zola-Morgan S. 1991 The medial temporal lobe memory system. Science 253: 1380–1386. 1896849 10.1126/science.1896849

[LM051821ZOTC41] Stark SM, Stevenson R, Wu C, Rutledge S, Stark CEL. 2015 Stability of age-related deficits in the mnemonic similarity task across task variations. Behav Neurosci 129: 257–268. 10.1037/bne0000055 26030427 PMC4451612

[LM051821ZOTC42] Stokes J, Kyle C, Ekstrom A. 2013 Complementary roles of human hippocampal subfields in differentiation and integration of spatial context. J Cogn Neurosci 27: 546–559. 10.1162/jocn PMC4312499 25269116

[LM051821ZOTC43] Treves A, Rolls ET. 1992 Computational constraints suggest the need for two distinct input systems to the hippocampal CA3 network. Hippocampus 2: 189–199. 10.1002/hipo.450020209 1308182

[LM051821ZOTC44] Treves A, Tashiro A, Witter ME, Moser EI. 2008 What is the mammalian dentate gyrus good for? Neuroscience 154: 1155–1172. 10.1016/j.neuroscience.2008.04.073 18554812

[LM051821ZOTC45] Tulving E. 1983 Elements of Episodic Memory. Oxford University Press.

[LM051821ZOTC46] Uncapher MR, Rugg MD. 2005 Encoding and the durability of episodic memory: a functional magnetic resonance imaging study. J Neurosci 25: 7260–7267. 10.1523/JNEUROSCI.1641-05.2005 16079408 PMC6725239

[LM051821ZOTC47] Vazdarjanova A, Guzowski JF. 2004 Differences in hippocampal neuronal population responses to modifications of an environmental context: evidence for distinct, yet complementary, functions of CA3 and CA1 ensembles. J Neurosci 24: 6489–6496. 10.1523/JNEUROSCI.0350-04.2004 15269259 PMC6729865

[LM051821ZOTC48] Wills TJ, Lever C, Cacucci F, Burgess N, Keefe JO. 2005 Attractor dynamics in the hippocampal representation of the local environment. Science 308: 873–876. 10.1126/science.1108905 15879220 PMC2680068

